# Qualification of the Low-pressure Cold Gas Spraying for the Additive Manufacturing of Copper–Nickel–Diamond Grinding Wheels

**DOI:** 10.1007/s11666-021-01291-y

**Published:** 2021-12-05

**Authors:** W. Tillmann, J. Zajaczkowski, I. Baumann, M. Kipp, D. Biermann

**Affiliations:** 1 Institute of Materials Engineering, TU Dortmund University, Dortmund, Germany; 2 Institute of Machining Technology, TU Dortmund University, Dortmund, Germany

**Keywords:** additive manufacturing, cold spraying, diamond grinding wheel, grinding, low pressure cold spraying

## Abstract

Grinding wheels are usually manufactured by powder metallurgical processes, i.e., by molding and sintering. Since this requires the production of special molds and the sintering is typically carried out in a continuous furnace, this process is time-consuming and cost-intensive. Therefore, it is only worthwhile for medium and large batches. Another influencing factor of the powder metallurgical process route is the high thermal load during the sintering process. Due to their high thermal sensitivity, superabrasives such as diamond or cubic boron nitride are very difficult to process in this way. In this study, a novel and innovative approach is presented, in which superabrasive grinding wheels are manufactured by thermal spraying. For this purpose, flat samples as well as grinding wheel bodies were coated by low-pressure (LP) cold gas spraying with a blend of a commercial Cu-Al_2_O_3_ cold gas spraying powder and nickel-coated diamonds. The coatings were examined metallographically in terms of their composition. A well-embedded superabrasive content of 12 % was achieved. After the spraying process, the grinding wheels were conditioned and tested for the grinding application of cemented carbides and the topographies of both the grinding wheel and the cemented carbide were evaluated. Surface qualities of the ground surface that are comparable to those of other finishing processes were reached. This novel process route offers great flexibility in the combination of binder and hard material as well as a cost-effective single-part and small-batch production.

## Introduction

For the production of customized and innovative tools for surface conditioning, there are special requirements (e.g., with regard to the coating thickness, adhesion, porosity, phase evolution and density) for MMC (metal matrix composite) coatings containing superabrasives, which cannot be achieved by conventional (hot) thermal spray processes. Cold gas spraying (CGS) provides a promising approach to deposit such coatings with the required characteristics and was therefore used in this study. In this process, compressed air or an inert gas is heated and expanded in a De Laval nozzle. Process temperatures typically range from room temperature up to about 800 °C, but CGS systems with higher process temperatures are also available on the market (Ref 1). The latter are mainly used for processing cermets such as WC-Co or high-strength nickel- or iron-based alloys. The spray materials are generally not melted. Rather, the comparatively low thermal energy is compensated by the deformation energy when the highly accelerated spray particles hit the substrate surface. The adhesion of individual splats is essentially based on the mechanical interlocking of the particles and on a phenomenon comparable to cold welding (Ref 2), although the specific mechanisms are still under discussion (Ref 3-5). If the kinetic energy of the particles is too low, no coating is formed. In this case, the spray particles, which are accelerated insufficiently, bounce off the surface. If the kinetic energy in turn is too high, the erosion speed is reached and, comparable to grit blasting, unwanted material removal of the substrate or coating material occurs. The kinetic energy or the velocity above which layer formation is possible is referred to as the “critical velocity” (Ref 1). For each material pairing of spray material and substrate material, different velocity windows exist in which a coating is formed.

A fundamental advantage of cold gas spraying is the low thermal energy transfer to the spray material (powder) and the substrate material. This means that even thermally sensitive materials can be sprayed or coated without notable thermal reactions. In addition, the equipment, especially in the area of low-pressure cold gas spraying (LP-CGS), is low priced and the investment and operating costs are low. Furthermore, high coating thicknesses can be realized due to the low thermally induced residual stresses. Since the lateral extent of the spray jet is narrow compared to other thermal spray processes due to the low spray jet divergence, a local coating of component areas can be realized. Due to the above-mentioned properties, cold gas spraying is regarded as an upcoming process in the field of additive manufacturing (Ref 6).

The basic feasibility of processing of hard material-metal powder blends by means of thermal spray processes can be found in the available literature (Ref 7-9). In previous investigations, detonation flame spraying was initially used for production of diamond-coated tools for rock machining (Ref 10-12). With this method, it was possible to embed large diamonds with a particle size range of 300-420 μm (40/50 USmesh) in a copper matrix. However, the diamond content was lower (at 2 vol.-%) compared to conventionally manufactured diamond-metal segments (powder metallurgical production, up to 10 vol %). As a further development, high-velocity oxygen-fuel spraying (HVOF) for the application of diamond-metal composite coatings was investigated and modeled (Ref 13-15). The HVOF process showed great potential for the application of diamond–nickel composite coatings with small, nickel-coated diamond grains (8-12 µm). Larger diamonds (40-60 μm grain size) could only be embedded in the coating with very low deposition efficiencies. Nevertheless, a coating is formed from the nickel, leading to the assumption that the large diamonds bounce off the surface while the molten nickel forms a coating on the surface. In addition, the HVOF process provided a low overall deposition efficiency (diamond and binder). For small diamond grit sizes, the nickel coating is sufficient to form a load-bearing coating with a diamond content of 33.5 ± 2.5 vol% (Ref 13). Measurements by x-ray diffraction analysis showed that the nickel coating protects the diamond from graphitization or decomposition. However, NiO was detected in the microstructure of the spray coating, which is formed by the atmospheric process conditions. In extended wear tests (pin-on-disk, ASTM G99), it could be demonstrated that the coating has properties corresponding to a grinding tool, based on the massive counterbody wear (Al_2_O_3_ ball) (Ref 15). The thickness of the produced diamond composite coatings is in characteristic coating thickness ranges of 300-400 µm for HVOF processes. Thicker coatings are not feasible due to the inherent residual stresses resulting from the process, which would result in delamination of the coating.

To achieve a higher coating thickness, which is a necessary requirement for the additive manufacturing of grinding wheels used for surface finishing, a low-pressure cold gas spraying process was used in this study. A mixture of nickel-coated diamonds, which were synthesized by the high-pressure–high-temperature (HTHP) process, and a Cu-Al_2_O_3_-powder was applied with a LP-CGS spraying system to achieve a coating thickness of ~1.5 mm. The coatings were subsequently heat treated to improve the interfacial bond as well as the coating cohesion. The produced coatings were examined metallographically regarding their composition by means of light microscopy and SEM/EDS as well as nondestructively by means of computer tomographic microscopy.

Additionally, tests concerning the dressing process of the novel grinding tools and grinding tests using cemented carbide as workpiece material were conducted. This approach makes it possible to assess whether the additively manufactured grinding wheels can withstand the stresses of a conditioning as well as a grinding process and have the potential to achieve surface qualities that are comparable to those of conventional grinding processes.

This novel approach makes it possible to manufacture grinding wheels economically in single-part or small-batch production. This enables the production of tools that are individually adapted to the respective application with regard to the grain size of the hard materials or binder content.

## Experimental

To obtain a homogeneous powder mixture, the two powders (nickel-coated synthetic HTHP diamonds (Ni-C, 8-12 μm grain size, CM-M Ni60, Ceratonia GmbH & Co. KG, Germany) as well as a commercial, LP-CGS-optimized Cu-Al_2_O_3_-powder blend (K-01-01, Dycomet Europe B.V., Netherlands)) were mixed in a weight ratio of 1:2 in a 3D shaker mixer (T2C, WAB AG, Germany) for one hour. The powder mixture was then analyzed by means of a field emission scanning electron microscope (JSM 7001F, Jeol Ltd., Japan) utilizing a backscattered electron detector (BSD) as well as electron dispersive spectroscopy (EDS). Furthermore, the two base powders as well as the powder blend were investigated by laser diffraction particle size analysis (S3500, Microtrac Retsch GmbH, Germany).

To evaluate the coating microstructure and the effect of the heat treatment following the coating process, the end faces of round aluminum samples (Ø40 x 6 mm, material AlSiMg0.5 / EN AW-6060) were pretreated for the coating process by grit blasting with EKF-40 corundum (grain size 355-500 µm) at a pressure of 4 bar and a distance of 100 mm at an angle of 45 °. Subsequently, the samples were cleaned in an ethanol ultrasonic bath for 10 minutes. The outer cylinder surfaces of the aluminum grinding wheel blanks (inner diameter 20 mm, outer diameter 87.5 mm, width 10 mm for the first wheel used in the dressing test; inner diameter 20 mm, outer diameter 80 mm, width 10 mm for the second wheel used in the grinding test) were prepared by the same process.

**Fig. 1:**
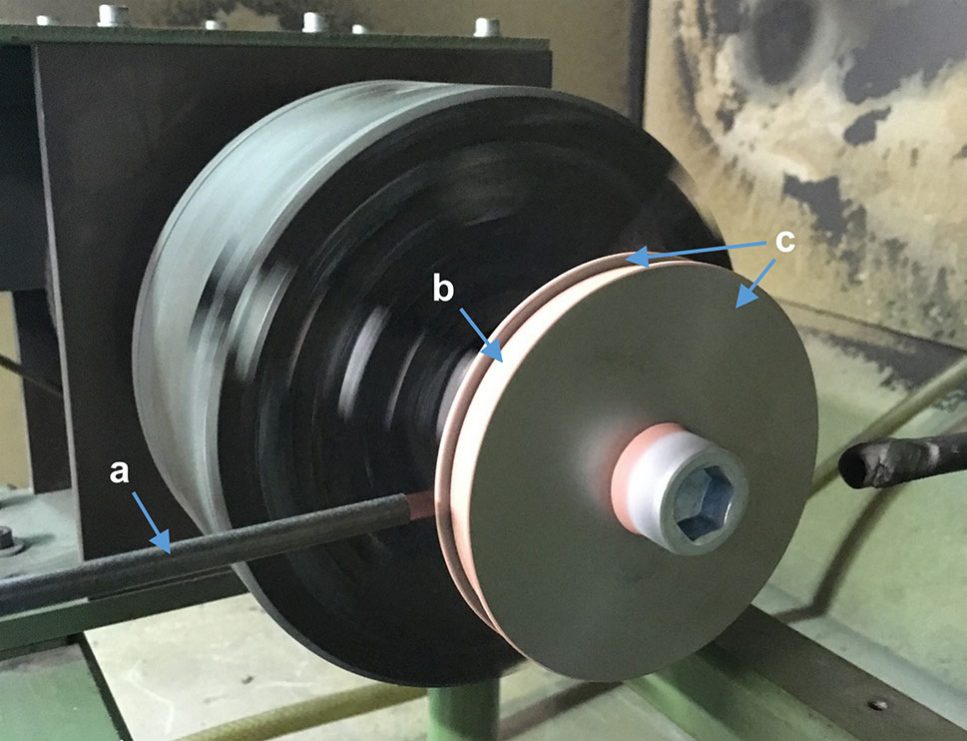
Spraying setup for the grinding wheel blank: (a) spraying nozzle; (b) substrate; (c) flanges

Before clamping onto the lathe, the end faces of the grinding wheel blanks were covered with flanges to prevent the coating from beveling toward the edge due to gas flow effects and to achieve a uniform coating result (Fig. 1). The grinding wheel blank was then rotated at a speed of 43.7 rpm to achieve a surface speed of 12,000 mm/min. The LP-CGS spray gun (DYMET 413, Dycomet Europe B.V., Netherlands) was moved tangentially at a distance of 15 mm at a speed of 43.7 mm/min using an industrial robot (type IRB 4400-60/1.95, ABB Ltd., Switzerland). For the flat samples, the coating was applied with a meandering path with a track pitch of 1 mm and a tangential speed of 12,000 mm/min. The gas inlet pressure was 6 bar, and the temperature setting was set to level 3 (corresponding to ca. 400 °C gas temperature at the nozzle inlet). The coating parameters can be found in Table 1.

**Table 1:** Parameters used for the LP-CGS process

Parameter	Value
Temperature	Setting 3 (~400 °C)
Gas inlet pressure	6 bar
Process gas	Compressed air
Powder feeder setting	3
Stand-off distance	15 mm
Surface speed	12,000 mm/min
Track pitch	1 mm
Number of passes	5

In order to achieve a material bond in the form of a diffusion zone and thus to improve the coating adhesion, a heat treatment process was performed subsequently to the coating deposition. Diffusion calculations were carried out with the Thermo-Calc DICTRA software to design the heat treatment process. A moderate temperature of 300 °C was selected as process temperature in order to reduce the formation of brittle intermetallic compounds such as AlCu_2_. Based on the results of the diffusion calculations, a heat treatment process at 300°C for 24 h was carried out in an atmospheric furnace (N100, Nabertherm GmbH, Germany).

The flat specimens were then used to prepare cross sections, which were subsequently examined by optical microscopy (BX51M, Olympus K.K., Japan), scanning electron microscopy (SEM) and energy-dispersive x-ray spectroscopy (EDS). Furthermore, smaller samples were cut from a flat specimen in the as-sprayed condition as well as from one in the heat-treated condition and nondestructively analyzed using an x-ray microscope (XRadia 520 Versa, Carl Zeiss AG, Germany) with a maximum voxel resolution of 700 nm. A total of 2400 images taken with a tube current of 140 kV, a power of 8 W and an exposure time of 10 s were combined to get the CT scans.

**Fig. 2:**
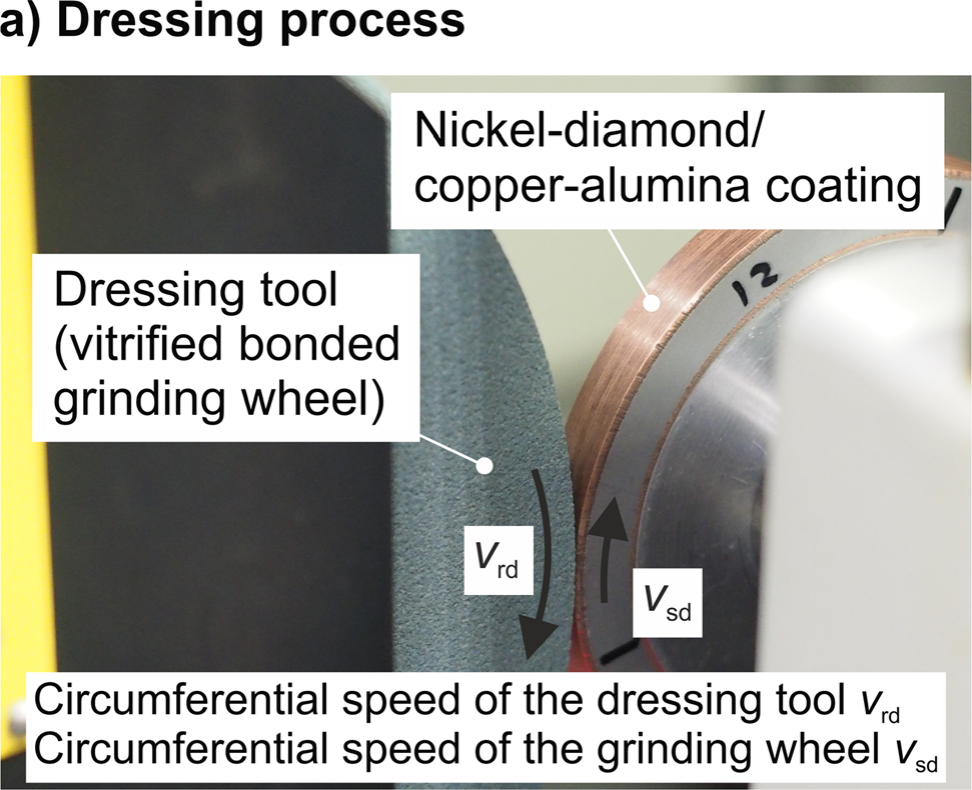
Conditions of the dressing process

Nickel-coated diamonds with a grain size of *d*_g_ = 8-12 µm are embedded in the grinding layer produced by the low-pressure cold gas spraying process. Consequently, the tools are intended to be tested in a finishing operation. Due to the high coating thickness of about 1.5 mm, it is possible to conduct a dressing process of the grinding tools, which is necessary to generate the required runout properties and to prepare the profile. These processes were conducted on a dressing machine type AP-800 Fusion (Rudolf Geiger Maschinenbau GmbH). The dressing process was realized using a vitrified bonded silicon carbide grinding wheel (grain size F240) as a rotating dressing tool, which is a common process for the dressing of diamond grinding wheels (Ref 16). Figure 2 shows the setup of the dressing process.

A substantial influencing variable in dressing processes with rotating dressing tools is the ratio of the dressing speeds *q*_d_ (Ref 17). This was set to *q*_d_ = - 0.8. Moreover, the overlapping rate in dressing was determined as *U*_d_ ≈ 15 and the depth of dressing cut was *a*_ed_ = 2 µm. As a result of 25 dressing swings, the total depth of the dressing cut was *a*_ed,ges_ = 50 µm. Emulsion was used as cooling lubricant for the dressing processes. Dressing conditions are summarized in Table 2. The tests conducted concerning the dressing processes focus the analysis of the grinding wheel topography. To this end, the grinding layer was analyzed using an optical measurement device (Alicona InfiniteFocus G5). The topography was examined at different levels of detail on six positions around the circumference of the grinding tool.

**Table 2:** Dressing conditions

Parameter	Value
Ratio of dressing speeds *q*_d_	–0.8
Overlapping rate *U*_d_	15
Depth of dressing cut *a*_ed_	2 µm
Dressing swings	25
Cooling lubricant	Emulsion

In a further step, one grinding wheel with a nickel–diamond/copper–alumina coating as grinding layer was tested in grinding processes, which were conducted on a tool grinding machine type *305micro* (Alfred H. Schütte GmbH & Co. KG). Cemented carbide (WC-Co, 12 % Co) with a grain size of 0.5 µm was selected as the workpiece material. Due to the comparatively small diamond grains, the surface was pre-ground using a diamond-grinding wheel with a grain size of D64 for the examinations concerning the surface roughness. During the grinding tests, the circumferential speed of the grinding tool was set to *v*_s_ = 15 m/s and the feed rate was *v*_f_ = 50 mm/min. The depth of the cut was varied in two steps and is specified as *a*_e_ = 10 µm and *a*_e_ = 20 µm. A summary of the grinding parameters is given in Table 3. In order to examine whether a material removal is achieved and to what extend the surface topography is affected, tactile roughness measurements were conducted. In addition, a white light microscope (NanoFocus µsurf) was used to analyze the surface topography of the ground cemented carbide in further detail.

**Table 3:** Grinding conditions

Parameter	Value
Circumferential speed *v*_s_	15 m/s
Feed rate *v*_f_	50 mm/min
Depth of cut *a*_e_	10 µm; 20 µm
Grinding strategy	Down grinding

## Results and discussion

**Fig. 3:**
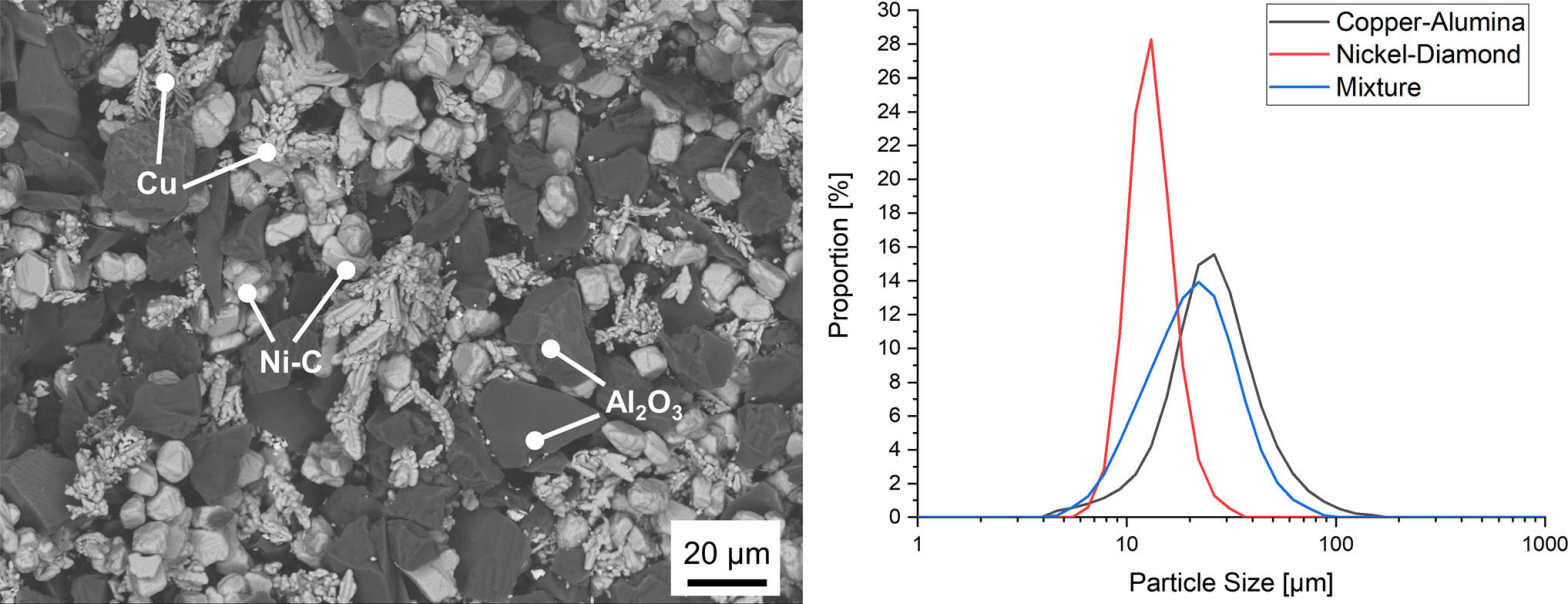
SEM-Image (BSD, 500x magnification) of the Cu-Al_2_O_3_-(Ni-C)-powder blend (left); laser diffraction analysis of the powders and the mixture (right)

After the tumbling mixing process, the powder blend shows a homogeneous distribution of the individual components in the SEM images (Fig. 3). It can be seen that the copper powder is in a dendritic morphology, which is caused by the electrochemical manufacturing route. This dendritic morphology is an excellent prerequisite for embedding other particles in the cold sprayed coating that might otherwise not adhere in the coating, since the “arms” of the dendrites fragment during the spraying process and interlock well with other particles (Ref 18). The alumina particles can be seen as larger, sintered and broken particles, which are included in the powder blend to further densify the coating by means of a shot peening effect/mechanical hammering of the applied coating as well as to prevent nozzle clogging by abrading sticking material in the injector/nozzle during the low-pressure cold spraying process (Ref 19). The laser diffraction analysis shows a narrow particle size distribution for the nickel-coated diamonds (d_10_ = 8.8 µm, d_50_ = 11.8 µm, d_90_ = 16.6 µm) and a broader distribution for the copper–alumina powder (d_10_ = 12.5 µm, d_50_ = 23.3 µm, d_90_ = 44.1 µm). The powder blend shows a similar particle size distribution to the copper–alumina powder with slightly lower values for the characteristic diameters (d_10_ = 9.5 µm, d_50_ = 18.9 µm, d_90_ = 34.8 µm).

**Fig. 4:**
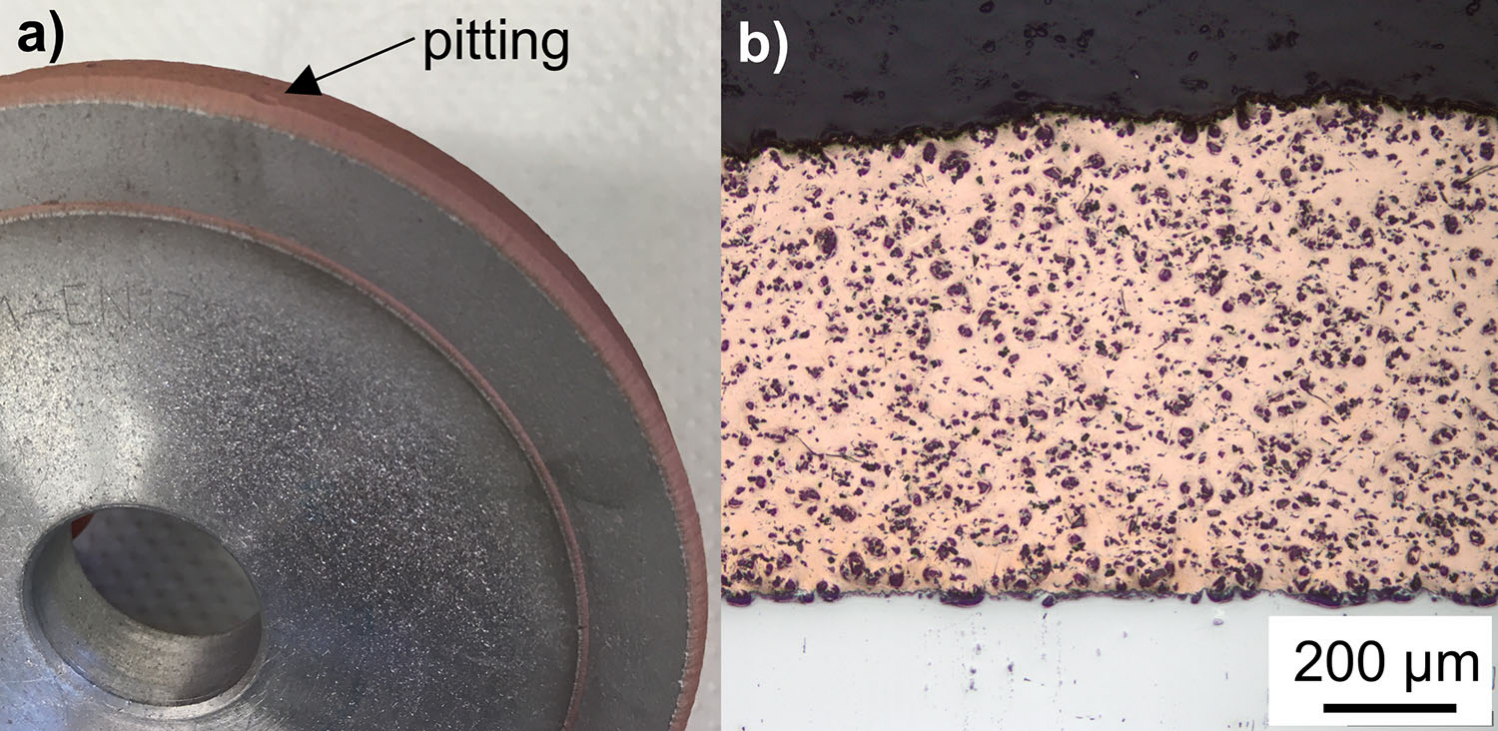
(a) Coated grinding wheel body; (b) light microscopy image of cross section

Figure 4 shows a macroscopic image of the coated aluminum base body. It can be clearly seen that a uniform, thick coating could be applied with only minor macro defects in the form of pittings, which can be attributed to smaller particles being diverted due to the bow shock effect (Ref 20, 21). The light microscope images of a cross section of a flat sample in Fig. 4b show a largely defect-free coating without any discernible cracks or pores. Slight inhomogeneities in the hard material distribution can be seen in the micrograph (Fig. 4b). This indicates that a segregation process has taken place during the powder feeding process, leading to an uneven distribution.

**Fig. 5:**
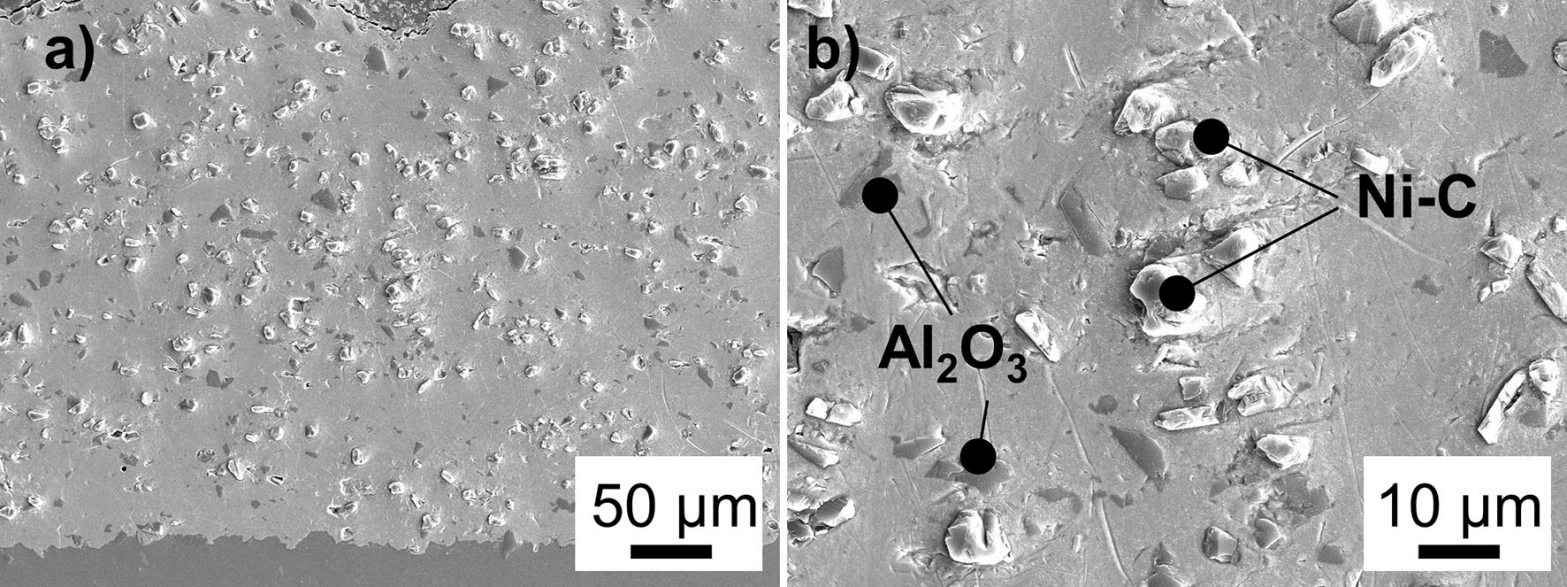
SEM-Images of cross section; (a) 100x magnification; (b) 2000x magnification

The SEM images show good embedding of the nickel–diamond particles in the copper matrix (Fig. 5b). An image-analytical evaluation using the software framework ImageJ of a total of 5 cross section images (one being shown in Fig. 5a) further shows that the volumetric fraction of nickel–diamond particles is much lower than in the original powder blend with a value of only 12.5 % compared to the original volumetric fraction of 28 % in the blend. This suggests a low deposition efficiency for these particles and points to further optimization potential of the spray parameters for this powder blend.

**Fig. 6:**
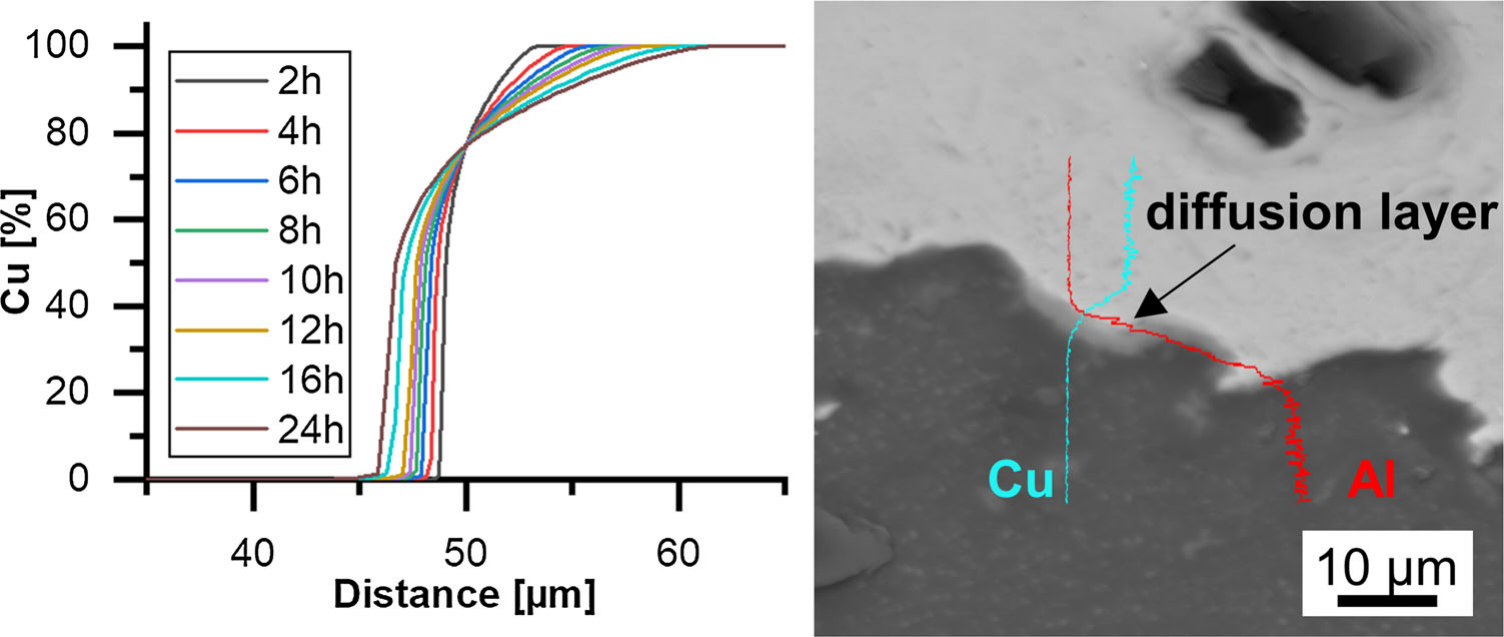
Calculated (Thermo-Calc DICTRA) and observed (SEM/EDS) diffusion zones at the Al-Cu interface

The numeric calculations of the diffusion process at the coating–substrate interface (Cu-Al) using the Thermo-Calc DICTRA software suggested that a diffusion layer with a width of about 15 µm should result after a 24-h-long heat treatment process at a temperature of 300 °C (Fig. 6a). However, in the SEM/EDS analysis, only small (~1 µm), very localized diffusion zones could be found at the Al-Cu interface (Fig. 6b), suggesting that insufficient bonding and breakup of the naturally and quickly formed Al-oxide layer has occurred during the cold spraying process. It is assumed that much higher process gas pressures could overcome this problem, which, however, is not achievable with the used low-pressure cold gas spraying system.

**Fig. 7:**
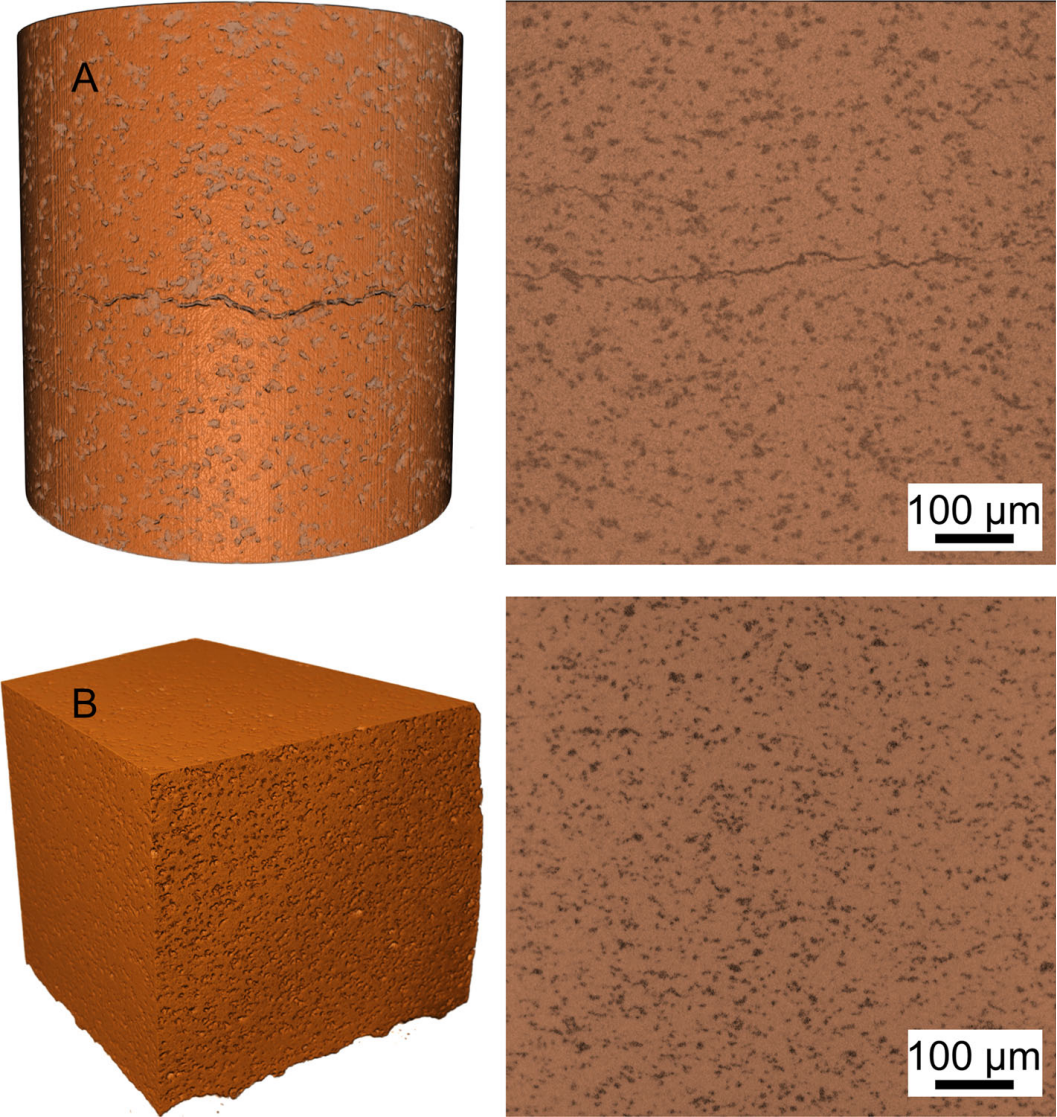
Computed tomographic images of a section of the generated nickel–diamond/copper–alumina coating in the as-sprayed condition (a) and in the heat-treated condition (b)

The nondestructive testing shows a large crack through the center of the sample in the as-sprayed condition (Fig. 7a), which can be detected throughout the whole CT scan area. Since these cracks primarily spread between the hard material particles (aluminum oxide or diamond), it is assumed that these particles significantly weaken the coating cohesion. Because these CT images were acquired in the as-sprayed condition (without heat treatment), the brittle behavior of the copper matrix, which has been extensively examined in other works (Ref 22, 23), also has a major influence on the cohesive strength of the coating. Further investigations with three heat-treated samples (one being shown in Fig. 7b) show no such major defects, suggesting that the heat-treated copper matrix shows a much more ductile behavior.

**Fig. 8:**
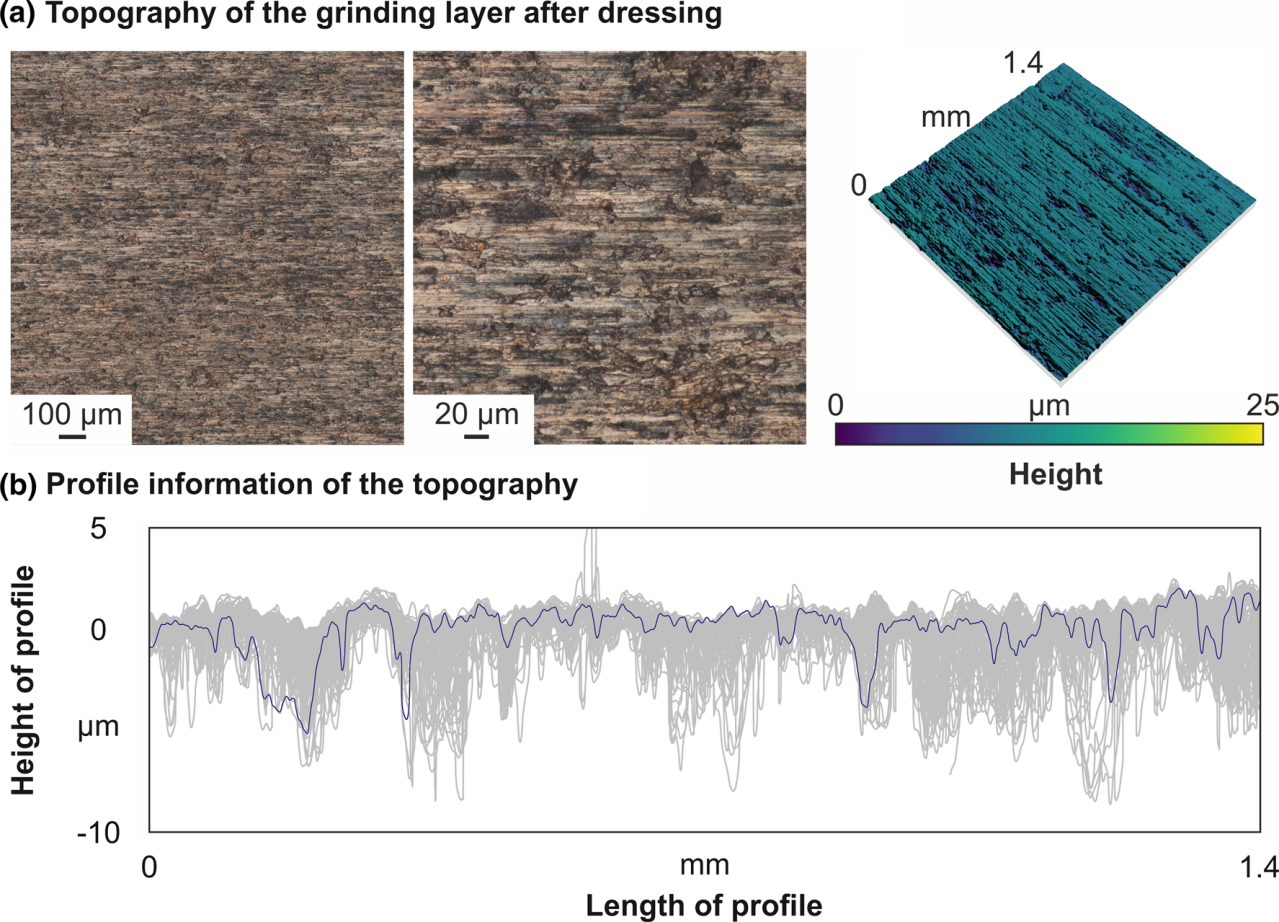
Topography of the grinding layer after dressing

Figure 8 shows the topography of the grinding layer after the dressing process. Next to microscopic images in two different magnifications, the grinding tool surface is also shown with additional height information. After dressing, the nickel–diamond/copper–alumina coating shows a quite homogenous surface structure with small grooves and some small dimples. The three-dimensional picture illustrates this in more detail. During the dressing processes, spalling of the nickel–diamond/copper–alumina coating was not detected. Therefore, on the basis of the first tests, it can be concluded that conventional dressing processes are suitable for the conditioning of the novel grinding layers as well.

**Fig. 9:**
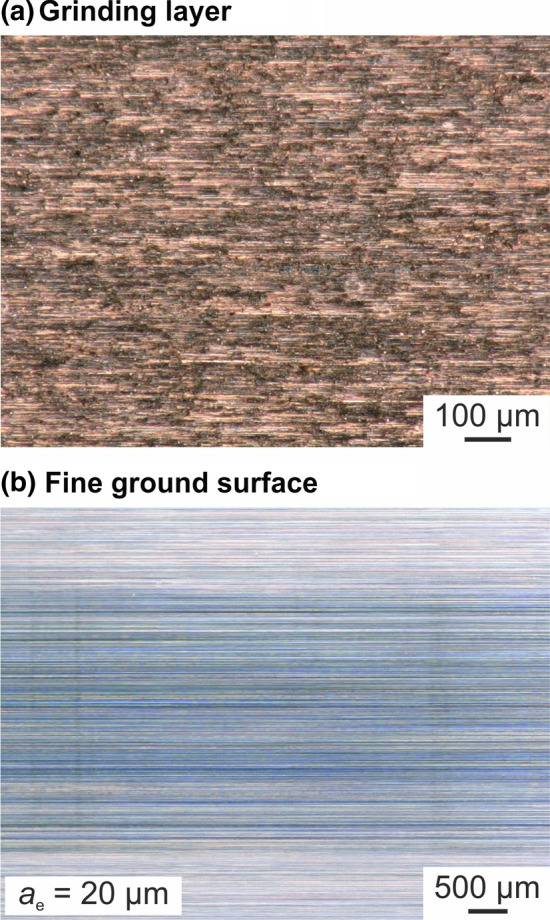
Overview of the grinding layer after grinding process and of the ground surface of the cemented carbide

With regard to the following grinding process, the change of the surface roughness resulting from the impact of the novel grinding layer was examined. To this end, Fig. 9 shows microscopic pictures of the sprayed coating after the grinding process and of the ground surface. No spalling of the grinding layer could be detected during grinding under the conditions mentioned. The picture of the fine ground surface reveals an impression of the altered surface area caused by the nickel–diamond/copper–alumina coating. Due to the fine grain size, it is possible to create a smooth surface topography with reflective surface properties. Nonetheless, the uniformity of the material removal in combination with the grinding wheel profile should be investigated in further investigations.

**Fig. 10:**
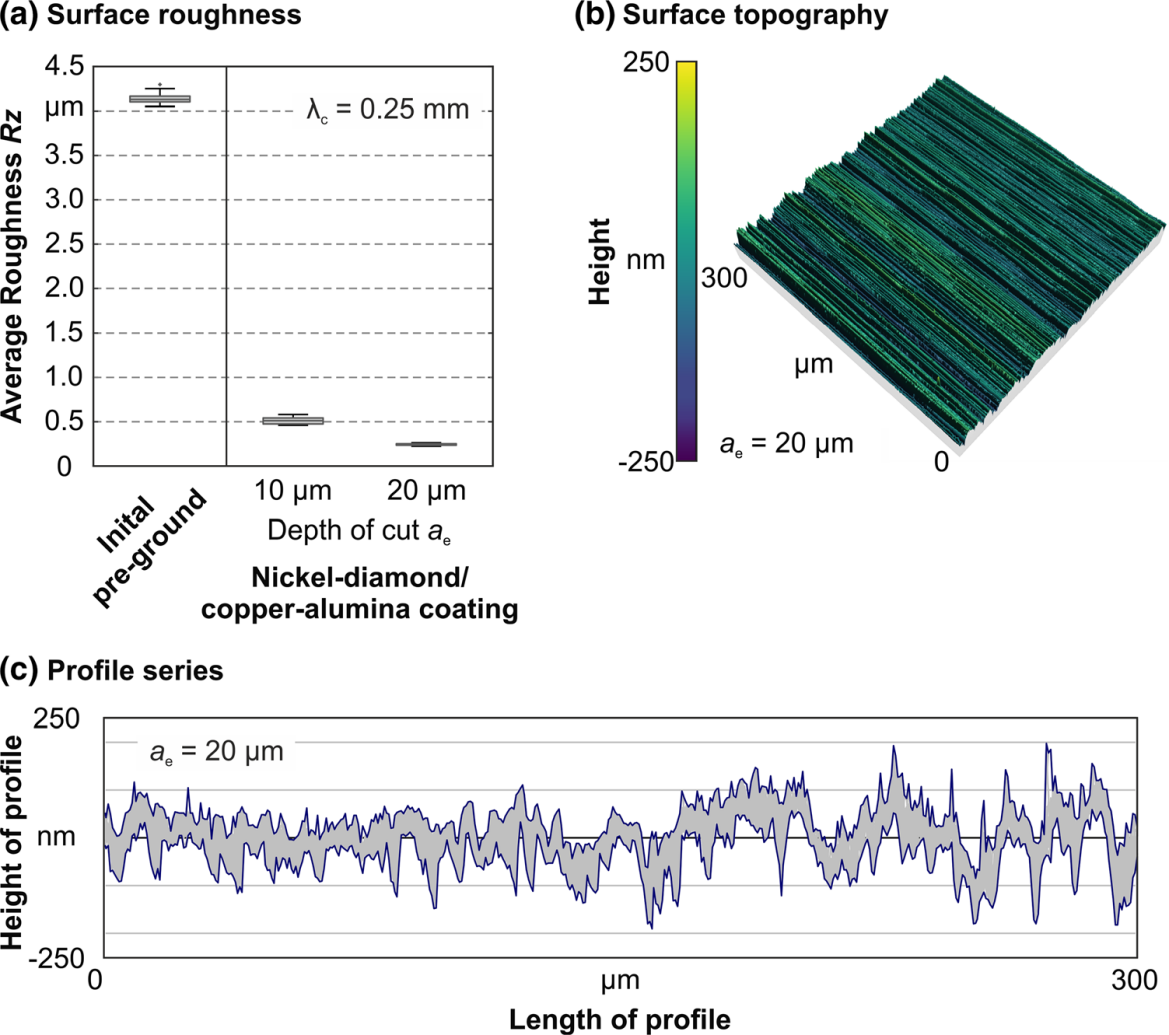
Roughness and topography of the ground surface of the cemented carbide

**Fig. 11:**
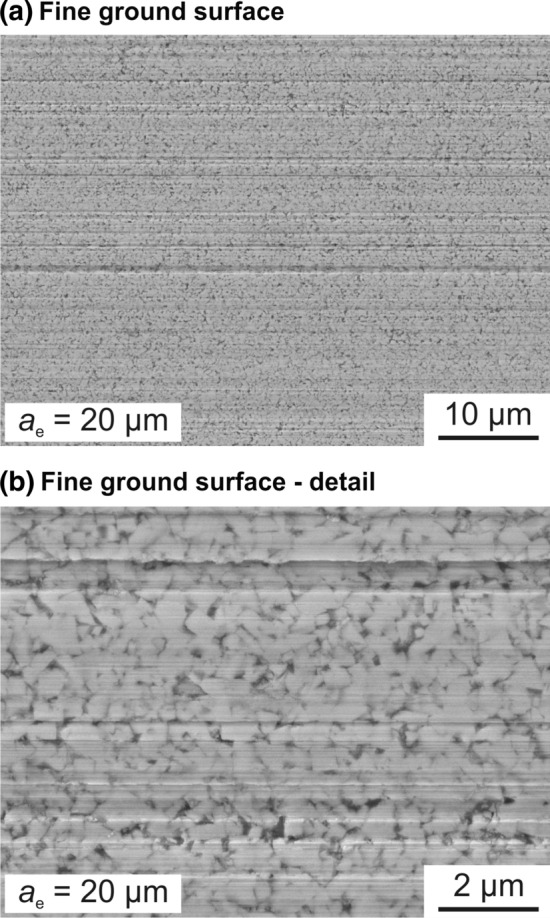
SEM images of the ground surface of the cemented carbide

To analyze the surface of the ground cemented carbide specimen in further detail, Fig. 10 provides information about the surface roughness and the surface topography. The surface roughness is quantified using the average roughness *Rz*. In the initial state, the ground surface shows an average roughness of *Rz* = 4.1 µm. Due to the finishing process, the surface roughness could be reduced to *Rz* = 0.24 µm for a depth of cut of *a*_e_ = 20 µm. The surface topography is typical for grinding processes, which becomes obvious due the linear scratches. In particular, this is apparent in Fig. 10(b) and (c). To gain further information about the material separation process when applying the nickel–diamond/copper–alumina coating, Fig. 11 shows SEM images of the ground surface. The surface exhibits a homogenous topography with some distinct scratches, which might occur due to specific protruding grains in the grinding layer. Larger areas characterized by breakouts were not observed. The results concerning the surface roughness are comparable to research in the field of fine finishing processes for cemented carbides. Within the scope of the first grinding tests, a surface roughness in the range of other abrasive fine finishing processes could be achieved (Ref. 24).

**Fig. 12:**
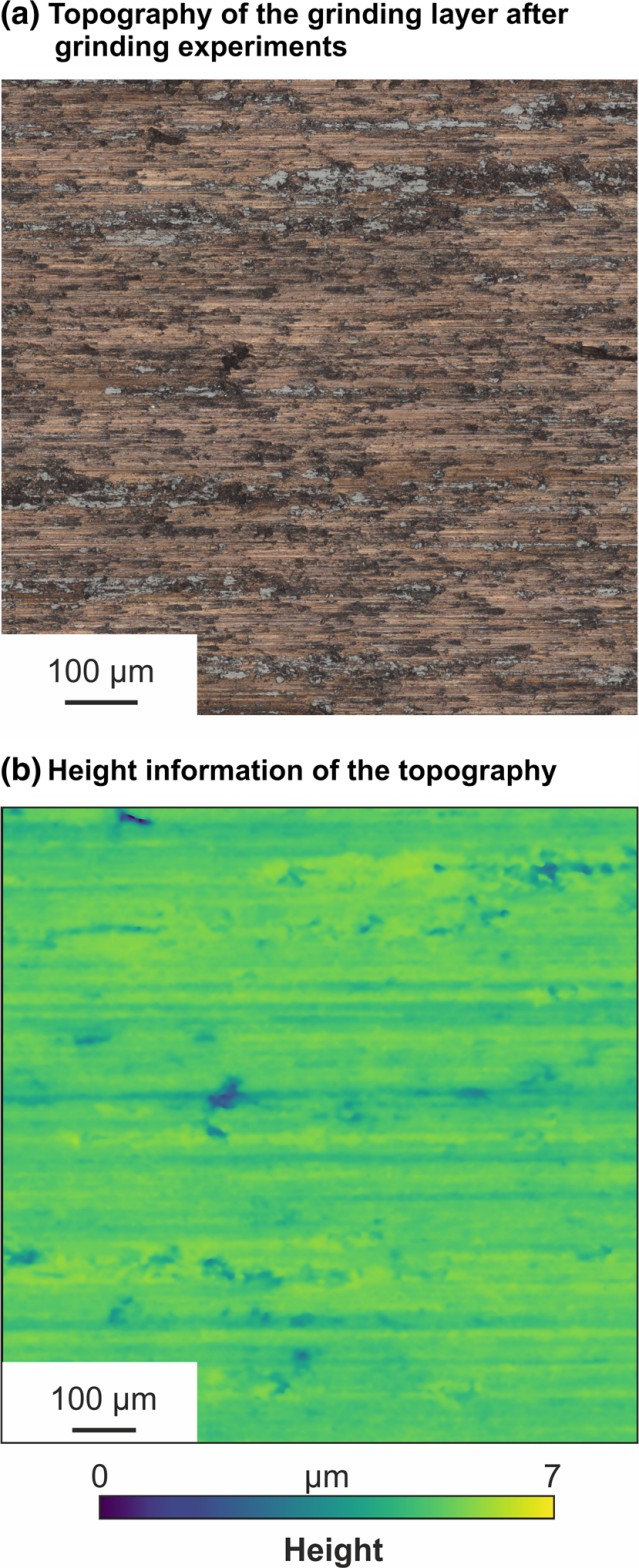
Microscopic images of the grinding layer after grinding experiments

With respect to the wear of the grinding wheel, microscopic images of the nickel–diamond/copper–alumina coating after grinding tests with varying material removal (a_e_ = 20 µm) are shown in Fig. 12. Some material debris adheres on the surface of the grinding layer. Nonetheless, the bonding still holds the abrasive grains. In further investigations, the characteristics of wear depending on process parameters as well as the durability of the grinding tools should be taken into account. Therefore, macroscopic wear as well as microscopic wear of the abrasive grains should be considered.

## Conclusion

A powder blend of nickel-coated synthetic diamonds with a commercial copper–alumina low pressure cold spray powder was deposited on aluminum blanks by means of LP-CGS. A coating with a thickness of approximately 1.5 mm could be successfully applied on the aluminum substrates, which allows the additively manufactured tools to be subsequently dressed. Microscopic images showed that the diamonds were well embedded in the thick spray coating. However, the coating contained a high proportion of aluminum oxide particles, which are undesirable for the use of the grinding tool.

Heat treating the coating system resulted in a material bond between the copper-based coating and the aluminum substrate in the form of a diffusion layer. However, this layer is very thin and could only be found localized, which is attributed to the oxide layer on the aluminum substrate, which was not completely removed by the cold gas spraying process.

The first investigations concerning the dressing of a nickel–diamond/copper–alumina coating in the application as grinding layer show that common dressing strategies using silicon carbide dressing tools are a suitable solution for the conditioning of the grinding wheel. Moreover, the first grinding tests provide promising results with regard to the achievable surface roughness. Nonetheless, the first tests reveal a high research potential especially concerning the process and wear behavior of the novel grinding layers.

Future studies should investigate the possibility of embedding pure hard material particles in a pure copper matrix, e.g., by means of high-pressure cold gas spraying, in order to be able to produce a tool with more homogeneous properties promising a more predictable behavior. Furthermore, a separate injection of both the hard material and the matrix material in the cold spray gun should be considered to prevent a segregation during the powder feeding process. Moreover, future research should focus on the central question whether higher grain sizes are processable with the low-pressure or high-pressure cold gas spraying processes, in order to gain further information in the field of conventional grinding processes. Regarding the dressing process, questions concerning the influence of varying dressing strategies on the topography of the nickel–diamond/copper–alumina coating and profile arise.
